# Unipedicular versus bipedicular percutaneous vertebroplasty for osteoporotic vertebral compression fractures: a prospective randomized study

**DOI:** 10.1186/s12891-015-0590-6

**Published:** 2015-06-14

**Authors:** Liang Zhang, Zhongjun Liu, Jingcheng Wang, Xinmin Feng, Jiandong Yang, Yuping Tao, Shengfei Zhang

**Affiliations:** Department of Orthopedics, Clinical Medical College of Yangzhou University, Subei Peoples Hospital of Jiangsu Province, No.98 Nantong West Road, Yangzhou, 225001 China; Department of Orthopaedics, Peking University Third Hospital, Beijing, 100191 China

**Keywords:** Osteoporotic vertebral compression fractures, Unipedicular, Bipedicular, Vertebroplasty

## Abstract

**Background:**

Percutaneous vertebroplasty (PVP) typically involves conventional lower-viscosity cement injection via bipedicular approach. Limited evidence is available comparing the clinical outcomes and complications in treating osteoporotic vertebral compression fractures (OVCFs) with PVP using high-viscosity cement through unipedicular or bipedicular approach.

**Methods and design:**

Fifty patients with OVCFs were randomly allocated into two groups adopting unipedicular or bipedicular PVP. The efficacy of unipedicular and bipedicular PVP was assessed by comparing operation time, X-ray exposure time, incidence of complications, vertebral height restoration, and improvement of the visual analogue scale (VAS), Oswestry disability index (ODI) and Short Form-36 (SF-36) General Health Survey scores.

**Results:**

The mean operative and exposure time to X-rays in the unipedicular PVP group was less than that of the bipedicular group (p < 0.05). No statistically significant differences were observed in the VAS score, ODI score, SF-36 score, cement leakage rate or vertebral height restoration between the two groups (p > 0.05).

**Conclusion:**

Unipedicular and bipedicular PVP are safe and effective treatments for OVCF. Compared with bipedicular PVP, unipedicular PVP entails a shorter surgical time and lower X-ray irradiation.

## Background

Osteoporosis is the most common systemic disorder worldwide, characterized by low bone mass, altered bone microarchitecture and increased risk of fragility fracture [1, 2]. In the European Union, an estimated 22 million women and 5.5 million men are afflicted with osteoporosis, with an annual cost of fractures related to osteoporosis pegged at euro 37.0 billion, expected to increase by 25 % in 2025 [3]. The most common fragility fractures associated with osteoporosis are vertebral compression fractures (OVCFs), affecting 25 % of postmenopausal women and more than 200 million individuals worldwide [4]. OVCFs cause substantial pain and deformity, leading to disability and poor quality of life. The usual treatment of OVCF includes analgesics, external braces and physical therapy. However, a few patients may still complain of severe pain after conservative treatments and even show progressive collapse of the vertebral body and kyphosis with or without neurological deficit [5–7]. Various techniques of vertebral body augmentation have been developed in an effort to treat these refractory cases. Over the past few decades, percutaneous vertebroplasty (PVP) has gained popularity as the optimal treatment modality for OVCF. PVP provided rapid pain relief and long-lasting effects in a large case series and nonrandomized controlled trails [6, 8, 9]. Although relatively safe and effective, PVP is associated with complications including cement leakage, soft tissue damage, pedicle fracture, nerve injury and spinal epidural hematoma [10, 11].

The detection rate of cement leakage ranges from 7 % by x-ray to 82 % using computed tomography (CT) [12]. While most leakages are asymptomatic, serious complications of nerve root or spinal cord compression and pulmonary embolism cannot be ruled out [10, 11, 13]. Reducing cement leakage using vertebral venography, gel-foam embolization, and kyphoplasty resulted in inconclusive outcomes [8, 14, 15]. High-viscosity cements have been demonstrated to effectively reduce the risk of epidural and venous cement leakage, thereby improving overall clinical safety [16–19]. The standard technique is typically carried out using a bipedicular approach [18, 19]. In recent years, unipedicular PVP has been advocated, reducing the operating and radiation exposure time, lowering the risk of cement leakage and complications caused by vertebral pedicle puncture. In addition, it increased the cost-effectiveness of the procedure as well as clinical efficacy. To compare the efficacy and adverse effects of unipedicular PVP with that of bipedicular PVP using high-viscosity cement, we conducted a prospective randomized and controlled study in our hospital. To the best of our knowledge, there has been no such report published until now.

## Methods

### Patients and controls

The Institutional Review Board of our hospital (Clinical Hospital of Yangzhou University Institutional Review Board Committee) approved this study, and patients provided informed consent prior to the study. From November 2010 to October 2012, a total of 58 patients with OVCF adopting PVP with high-viscosity bone cement were included in our study. Clinical indications for vertebral augmentation were regularly confirmed by an interdisciplinary team of oncologists, radiation oncologists, spine surgeons, and interventional radiologists prior to the intervention.

Study inclusion criteria were as follows: (1) age over 50 years; (2) single-level OVCF; (3) focal back pain in the midline unresponsive to at least 8 weeks of conservative treatments; (4) back pain related to the location of the OVCF on magnetic resonance imaging (MRI) radiographs; (5) presence of an apparent bone edema in the fractured vertebra on T2-weighted short-tau inversion recovery sequences (STIR) in MRI; (6) bilateral pedicle intact without fracture; and (7) decreased bone mineral density (T scores < −1). We excluded patients with (1) vertebral compression fracture due to causes other than osteoporosis; (2) spinal cord compression or stenosis of the vertebral canal > 30 % of the local canal diameter; (3) neurologic deficits; (4) incurable bleeding disorders; (5) systemic or local spine infections; (6) severe comorbidity of heart, liver, kidney, and lung intolerance to surgery.

All eligible patients were assigned a serial number according to the consecutive sequence of hospitalization and allocated to group U (using unipedicular approach) or group B (using bipedicular approach) randomly by computer. The study population consisted of 24 patients in group U (8 male and 16 female, mean age = 69.2 years, range = 52–81 years) and 26 patients in the group B (10 male and 16 female, mean age = 70.5 years, range = 57–83 years). All procedures were performed by the same surgeon.

### Surgical technique

All surgical procedures were conducted under general anesthesia, with the patient in prone position on a carbon-fiber radiolucent, C-arm table. After localizing the fractured vertebra using fluoroscopy, the surgeon placed overlapping palm on the vertebral spinous process to push ventrally slowly and partially reduce the fractured vertebra. A small skin puncture was made approximately 2.5 cm off of mid-line. The introducer was directed to the junction of the middle and anterior 1/3 of the affected vertebral body under C-arm fluoroscopic guidance. Once the needle was in the optimal position, the high-viscosity polymethylmethacrylate (PMMA) bone cement was injected with a specially designed delivery system (Disc-O-Tech Medical Technologies Ltd. Herzliya, Israel) according to the manufacturer’s specifications. The injection procedure was carefully controlled under strict lateral fluoroscopy and stopped whenever epidural or paravertebral opacification was observed or the cement reached the dorsal quarter of the vertebral body.

### Outcome measurements

We emphasized both surgical complications and clinical outcomes in both groups three days and two years after surgery. Outcome measures included: (1) surgery time (from skin incision to suturing), exposure time to the X-ray C-arm machine and cement dosage; (2) Visual Analog Scale (VAS) score for analgesic efficacy evaluation; (3) Oswestry Disability Index (ODI) for functional assessment; (4) Short Form-36 General Health Survey (SF-36) for quality of life evaluation: Physical Component Summary (PCS) and the Mental Component Summary (MCS); (5) complications including cement leakage and adjacent vertebral fractures; and (6) radiographic outcomes including anterior and middle vertebral body height variation, (calculated by fractured vertebral body height/normal vertebral body height × 100 %). Normal heights of the vertebrae were defined as the mean of the equivalent values for the adjacent superior and inferior non-fractured vertebrae. Anterior and middle vertebral height was defined as the distance between the upper and lower endplates at the anterior and middle vertebral body wall and in the center of the vertebral body.

### Statistical analysis

Data were presented as the mean ± standard deviation. The SPSS for Windows Version 13.0 (SPSS, Chicago, IL) was used for the analysis. Statistical analysis was performed by an independent statistician blinded to the surgical procedures. The statistical significance of pre- and post-surgical clinical and radiographic data within each group and between groups was evaluated using independent *t*-test and chi-square test. The difference of cement leakage between two groups was assessed using the *χ*2 test. The level of statistical significance was set at p <0.05.

## Results

### Surgical parameters

The mean surgery time was 41.2 ± 5.2 min in group U, which was shorter than 55.7 ± 7.3 min in group B (*p* < 0.001) (Table 1). Patients were exposed to X-rays 33.7 ± 5.2 min in group U and 46.5 ± 6.6 min in group B, and the difference was statistically significant (*p* < 0.001) (Table 1). The bone cement volume was 3.1 ± 0.4 mL in group U and 5.0 ± 0.5 mL in group B, and this difference was also statistically significant (*p* < 0.001) (Table 1).

**Table 1:** Surgical parameters of the studied population

	Group U	Group B	*p-value*
	N = 24	N = 26	
Male/female	5:19	8:18	0.42^a^
Age (years)	71.7 ± 7.5	72.1 ± 6.0	0.84^b^
Operative time	41.2 ± 5.2	55.7 ± 7.3	0.001^b^
Exposed to X-rays time	33.7 ± 5.2	46.5 ± 6.6	0.001^b^*
Injected cement volume	3.1 ± 0.4	5.0 ± 0.5	0.001^b^*
Cement leakage	5	9	0.28^a^

Group U: PVP through unipedicular approach

Group B: PVP through bipedicular approach

^a^Pvalues for between-group comparison were determined by *χ*2 tests

^b^Pvalues for between-group comparison were determined by t tests

*Statistically significant (*p* value <0.05)

### Clinical outcomes

There was no difference between the 2 groups in terms of the pre-operative VAS, ODI, PCS and MCS measurements (Table 2). Patients in both groups exhibited marked and sustained pain reduction, function and quality of life improvement. Significant statistical differences were found before and after operation in terms of VAS, ODI and SF-36 scores (Table 2). However, no statistical differences were found 3 days or 2 years postoperatively between the 2 groups in terms of the clinical outcomes (Table 2).

**Table 2:** Clinical outcomes of the two groups

Time	Parameter	Group U	Group B	*p-value*
		N = 24	N = 26	
*Preoperative*	VAS	8.1 ± 1.0	8.0 ± 1.2	0.78
	ODI	45.2 ± 5.1	43.2 ± 4.1	0.13
	PCS	31.6 ± 4.9	29.1 ± 3.7	0.13
	MCS	31.2 ± 3.9	32.1 ± 3.1	0.41
*Three days*	VAS	2.6 ± 0.8*	2.5 ± 0.9†	0.73
*postoperative*				
	ODI	27.3 ± 4.2*	26.1 ± 3.4†	0.28
	PCS	44.8 ± 5.2*	42.6 ± 4.5†	0.11
	MCS	41.0 ± 3.8*	40.1 ± 4.2†	0.43
*Two years*	VAS	2.1 ± 0.9*	2.1 ± 0.7†	0.97
*postoperative*				
	ODI	19.9 ± 4.6*	18.9 ± 2.6†	0.26
	PCS	52.9 ± 3.7*	52.8 ± 5.5†	0.93
	MCS	45.7 ± 3.8*	45.3 ± 3.7†	0.74

Group U: PVP through unipedicular approach

Group B: PVP through bipedicular approach

VAS visual analogue scale; ODI oswestry disability index;PCS physical component summary; MCS mental component summary

*P* values for between-group comparison were determined by t tests

*P = 0.000, compared to Preoperative outcomes in Group U

†P = 0.000, compared to Preoperative outcomes in Group B

### Radiological outcomes

There were no surgical complications in either group. The bone cement leakage rate was 20.8 % (5 of 24) in the group U and 34.6 % (9 in 26) in group B, which was not a statistically significant difference (p = 0.28). Of the 5 bone cement leakages in group U, one occurred into the anterior vertebra, 2 were leakages into the disc and another 2 were venous leaks. Of the 9 bone cement leakages in group B, 3 occurred into the disc, one into paravertebral, 2 into epidural, and another 3 were venous leaks. However, no clinical symptoms were identified due to leakage and no special treatment was necessary.

The anterior and middle vertebral heights of the fractured vertebra before surgery showed no significant diffeence between the 2 groups (Table 3). Similarly, no statistical difference was found 3 days later or even after 2 years. However, significant statistical differences were found before and after the operation in terms of the anterior and middle vertebral heights of the fractured vertebra (Table 3).

**Table 3:** Radiological outcomes of the two groups

Time	Parameter	Group U	Group B	*p-value*
		N = 24	N = 26	
*Preoperative*	Anterior vertebral body	41.8 ± 7.5	42.9 ± 8.0	0.63
	height variation, %			
	Middle vertebral body	46.9 ± 5.4	47.3 ± 6.1	0.81
	height variation, %			
*Three days*	Anterior vertebral body	56.3 ± 6.4*	59.0 ± 5.2†	0.11
*postoperative*	height variation, %			
	Middle vertebral body	58.5 ± 4.8*	59.6 ± 5.5†	0.44
	height variation, %			
*Two years*	Anterior vertebral body	53.0 ± 5.9*	55.6 ± 5.3†	0.11
*postoperative*	height variation, %			
	Middle vertebral body	55.4 ± 5.0*	56.8 ± 5.4†	0.34
	height variation, %			

Group U: PVP through unipedicular approach

Group B: PVP through bipedicular approach

*P* values for between-group comparison were determined by t tests

*P = 0.000, compared to Preoperative outcomes in Group U

†P = 0.000, compared to Preoperative outcomes in Group B

## Discussion

PVP is the optimal treatment for OVCF and provides rapid pain relief and stabilization of the fractured vertebral bodies [8, 20, 21]. Although cement leakage is reported as high as 73 % for both PVP types, most leakages remain clinically asymptomatic, and even small quantities of leakage may have a significant clinical impact [12]. The three factors that may influence the cement flow into and out of the vertebral body include: bone and fracture-related parameters, cement properties, and injection methods. Although fracture morphology is impossible to control, the cement properties and method of injection may be manipulated to ultimately decrease the complication rate.

Viscosity of bone cement used in PVP is hypothesized to influence the outcome of the procedure in various ways. Increased viscosity leads to increased circularity of the cement cloud and decreased spreading distance [22, 23]. PVP with high-viscosity cement offers all the advantages, especially minimizing the risk of cement leakage and significantly increasing the clinical safety [22, 24]. In the PVP procedure, unipedicular approach is being increasingly used to treat OVCF, to reduce medical costs and X-ray exposure, as well as for better clinical efficacy [25, 26]. In our prospective, randomized study, both unipedicular and bipedicular PVP group achieved satisfactory results and patients’clinical outcome parameters were significantly improved and consistent compared to pre-surgical levels. The improvement in the clinical outcome and recovery of vertebral heights were not significantly different between the two groups under the strict inclusion criteria.

In theory, bipedicular PVP shows increased incidence of complications such as tissue trauma, nerve injury and pedicle fractures. However, in our study, no pedicle fractures or nerve damage occurred in either group. The complications resulting largely from poor operative technique can be minimized at the operator level.

Some clinical studies have positively correlated the bone cement injection volume with the leakage of bone cement [27]. In theory, the risk of bone cement leakage is also twice that of unipedicular approach. However the difference in cement leakage rates between unipedicular and bipedicular PVP with conventional viscosity cement was not statistically significant [28–31]. The cement leakages in PVP with high-viscosity cement are still unknown. A larger volume of injected cement through the bipedicular approach is also more likely to result in extravasations. The most common cement leakage was venous leakage (6.1 %) and intradiscal leakage (8.2 %) in PVP with high-viscosity Confidence bone cement reported by Anselmetti et al. [22]. However, the leakage rate was almost as high as 47 % reported by Georgy et al. [18]. The cement leakage rates in the above two studies were far different. By comparison, we found that the biggest difference may be the methods adopted (unipedicular approach by Anselmetti vs. bipedicular approach by Georgy) and cement leakages detected (CT by Anselmetti vs. plain film by Georgy). CT is now regarded as the gold standard to assess cement leakage [12]. Whether the lower cement leakage rate reported by Anselmetti was due to the different PVP is unclear. In this study, the cement leakage rates of both groups were lower but not statistically significant compared with PVP using standard low-viscosity cement. The results suggested that increased bone cement injection did not result in increased bone cement leakage rate, which may be attributed to the nature of high-viscosity bone cement itself.

The X-ray exposure and operation time of unipedicular approach were significantly reduced. Unipedicular PVP lessens the radiation dose, thereby reducing health risks to the medical staff performing the PVP. If 5 % of all vertebral compression fractures in the United States were treated by unipedicular kyphoplasty, instead of bipedicular kyphoplasty, the savings would be > $32 million per year [32]. Thus, our results support the concept that the unipedicular technique is a faster, cost-effective alternative that still provides a comparable correction of spinal deformity to the bipedicular technique. The unipedicular technique is specifically indicated for the elderly, or patients who have more than one affected vertebra.

The limitations of our study are related to the relatively small sample size and the follow-up of only three days and two years. We also failed to perform a direct comparison of vertebroplasty with kyphoplasty using high-viscosity cement. Further study using a larger sample size and longer or more frequent follow-ups are needed to confirm our results.

## Conclusion

Unipedicular and bipedicular PVP are safe and effective treatments for OVCF. Although the pain relief and improved physical ability were comparable with either technique, we encourage the use of the unipedicular approach as the preferred surgical technique for treatment of OVCFs due to less operation time, limited X-ray exposure, minimal cement introduction and extravasation.

## Abbreviations

PVP: Percutaneous vertebroplasty
OVCFs: Osteoporotic vertebral compression fractures
VAS: Visual analogue scale
ODI: Oswestry disability index
SF-36: Short Form-36
MRI: Magnetic resonance imaging
PMMA: polymethylmethacrylate
